# Self-shedding and sweeping of condensate on composite nano-surface under external force field: enhancement mechanism for dropwise and filmwise condensation modes

**DOI:** 10.1038/s41598-017-09194-1

**Published:** 2017-08-17

**Authors:** Jie Sun, Hua Sheng Wang

**Affiliations:** 1 School of Chemical Engineering and Technology, Xi’an Jiaotong University, Xi’an, 710049 China; 2 Institute of Engineering Thermophysics, Chinese Academy of Sciences, Beijing, 100190 China; 3 School of Engineering and Materials Science, Queen Mary University of London, London, E1 4NS UK

## Abstract

In this work, we propose the concept to use the hydrophilic or neutral surface for condensation heat transfer and to use the superhydrophobic surface for enhancement by self-shedding and sweeping of condensate. Molecular dynamics simulation results show that no matter the vapor condenses on the solid surface in dropwise or filmwise mode, the grown-up condensate self-sheds and falls off the superhydrophobic surface, sweeping the growing condensate on the condensing surface downstream. We characterize the dynamics of condensate that the continuous self-shedding and sweeping effectively remove the droplets from the solid surface in dropwise mode or thin the condensate film on the solid surface in filmwise mode, which significantly enhances the condensation heat transfer. We reveal that the mechanism for self-shedding is two-fold: (1) that the external force on condensate bulk defeats the adhesive force between the condensate and the solid surface triggers the self-shedding; (2) the release of the surface free energy of condensate promotes the self-shedding. We also reveal that the mechanism of heat transfer enhancement is essentially due to the timely suppression over the growing condensate bulk on the condensing surface through the self-shedding and sweeping. Finally, we discuss the possible applications.

## Introduction

Condensation is a common physical phenomenon that plays an important role in many applications (see refs 1 and 2 and references therein). When a vapor is in contact with a solid surface at some temperature below the saturation temperature of the vapor, the vapor condenses to liquid on the surface, releasing to the surface the energy difference between the vapor and liquid states. Surface condensation is conventionally categorized as either dropwise condensation (DWC) on non-wetting surface or filmwise condensation (FWC) on wetting surface, with the general understanding that dropwise mode being an order of magnitude more efficient in heat transfer than the filmwise mode^1, 3^. Therefore, it is highly desirable to utilize the dropwise mode in engineering applications to make systems more thermally-efficient and more compact. However, since DWC was recognized in 1930’s^1, 4^, it has been difficult in practice to sustain the dropwise mode for a sufficiently long time^1^.

With the fast-developing surface-nanomachining and surface-coating technologies, the surface wetting characteristics becomes artificially-customized^5–7^. Recently, increasing interest has been drawn to the potential use of highly-customized superhydrophobic surfaces to sustain DWC through spontaneous coalescence-induced droplet jumping^2, 8–13^. The superhydrophobicity is achieved by physically or chemically reducing the surface free energy, which leads to the coalescence-induced droplet jumping^14, 15^. It has been demonstrated from the molecular level that superhydrophobicity generally serves as a remarkable interfacial thermal resistance due to remarkably weak fluid-solid interaction (low surface free energy)^16–19^. In addition, the superhydrophobicity leads to very large contact angle (normally above 150°)^6^ and therefore reduces the effective heat transfer area (solid-liquid contact area)^20^. Considering these aspects, it is necessary to focus on not only the sustainability but also the heat transfer performance of DWC on superhydrophobic surface. On the other hand, alternative resorts of enhancing condensation heat transfer should be explored. In this work, we report a new method, using a composite nano-surface, to sustain and enhance condensation heat transfer under external force field and we reveal the microscopic mechanism.

## Results

**Figure 1:**
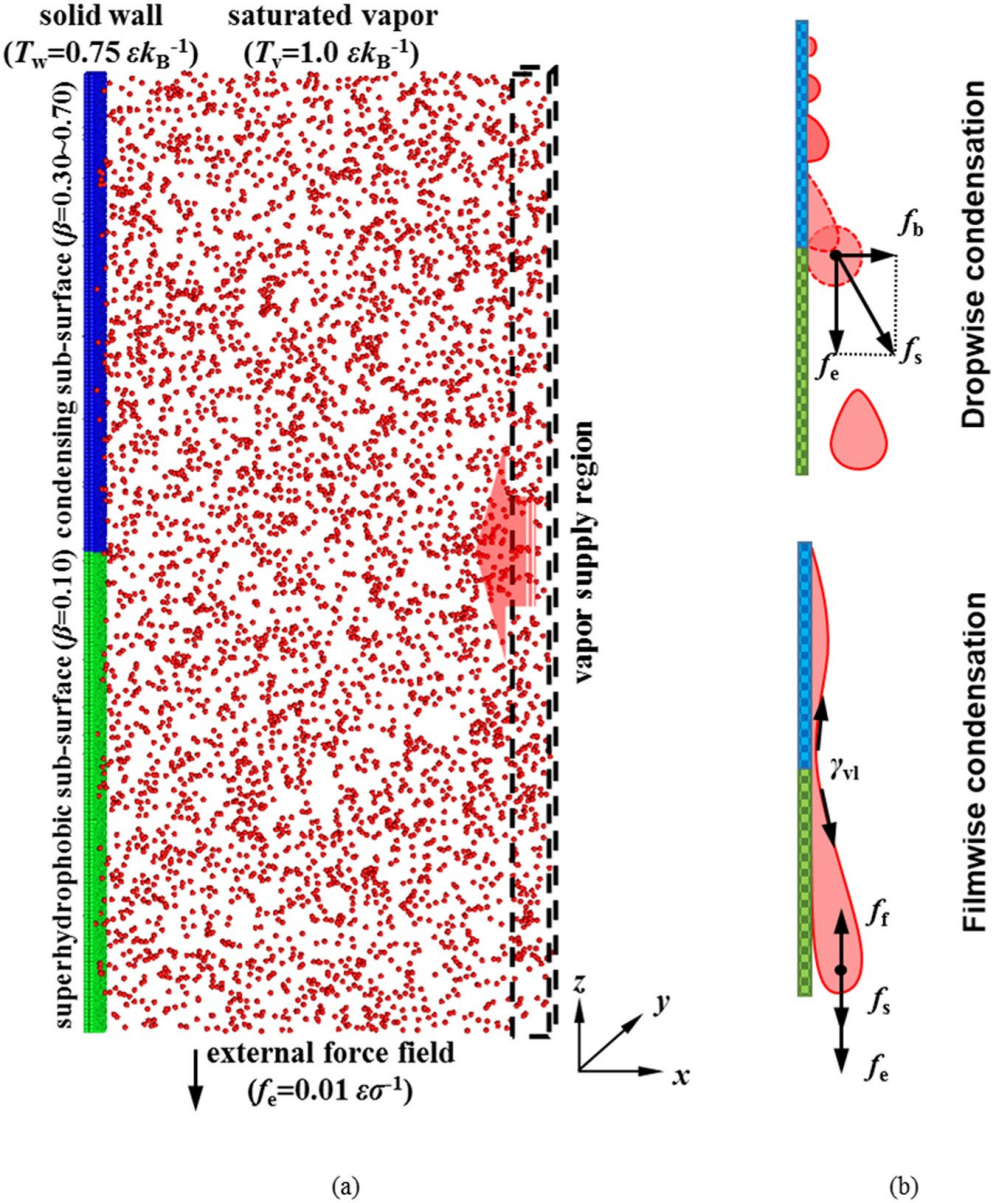
Schematic presentations of (**a**) computational model and (**b**) force analyses. The simulation size measures *l*_*x*_ × *l*_*y*_ × *l*_*z*_ = 98.2 *σ* × 8.5 *σ *× 97.7 *σ*. The saturated vapor is at ${T}_{{\rm{v}}}=1.0\,\varepsilon {k}_{{\rm{B}}}^{-1}$ (red) and the solid wall is at ${T}_{{\rm{w}}}=0.75\,\varepsilon {k}_{{\rm{B}}}^{-1}$. The vaper supply region is rightmost and the solid wall is leftmost, which is comprised of condensing surface with *β* = 0.30~0.70 (blue) and superhydrophobic surface with *β* = 0.10 (green). The external force field is ${f}_{{\rm{e}}}=0.01\,\varepsilon {\sigma }^{-1}$ in the *z-*direction. *f*_b_ is the bouncing force, *f*_f_ is the frictional force, *f*_s_ is the resultant shedding force and *γ*_vl_ is the liquid-vapor surface tension.

We use molecular dynamics (MD) simulation to carry out the investigation of condensation on vertically composite nano-surface (see Fig. 1). The fluid-fluid and fluid-solid interactions are governed by the Lennard-Jones (L-J) potential function, where the parameter *β* measures the relative strength of fluid-solid bonding. A small value of *β* means low solid surface free energy and hydrophobicity while a large value of *β* means higher solid surface free energy and hydrophilicity^17, 21–26^. The surface wettability is commonly interpreted by contact angle *θ*, a readily measureable quantity^27–29^. The relation between *θ* and *β* at thermal equilibrium state of $T=0.75\,\varepsilon {k}_{{\rm{B}}}^{-1}$ (*k*_B_ being the Boltzmann constant) is identified to be a monotonically decreasing function based on our recent investigations^18, 19^. In this work, *β* is chosen to be 0.10, 0.30, 0.35, 0.40, 0.45, 0.70 and the corresponding *θ* are 157.7°, 107.3°, 94.7°, 82.1°, 67.9°, 20.2°, respectively.

The vertical, composite nano-surface is arranged leftmost in the simulation box (see Fig. 1a). The upper half of the composite nano-surface is named as the condensing surface with *β* = 0.30~0.70 and the lower half is the superhydrophobic surface with *β* = 0.10. The dual-*β* surface is labeled as *β* = (0.30~0.70, 0.10). The periodic boundary condition is applied at the top, bottom and sides and the diffuse reflection boundary is applied at the rightmost end. An external force ${f}_{{\rm{e}}}=0.01\,\varepsilon {\sigma }^{-1}$ is exerted on each fluid molecule in the *z-*direction. All condensation simulations are carried out with solid surface temperature at ${T}_{{\rm{s}}}=0.75\varepsilon {k}_{{\rm{B}}}^{-1}$ and saturated vapor temperature at ${T}_{{\rm{v}}}=1.0\varepsilon {k}_{{\rm{B}}}^{-1}$, respectively.

We first investigate the dynamics of condensate on two typical surfaces with dual *β* (*β* = (0.35, 0.10) and *β* = (0.70, 0.10)) corresponding to DWC and FWC, respectively. The condensate is found to behave diversely in different condensation modes.

### Dropwise condensation on surface with β = (0.35, 0.10)

1. Nucleation and coalescence
  Clusters are seen to randomly deposit on the condensing surface. Some clusters are able to migrate and coalesce with other clusters (see Fig. 2a at *t* = 1000 *τ*). After plentiful coalescences, a primary droplet emerges (see Fig. 2a at *t* = 2000 *τ*). As the primary droplet grows, the vertically-driving force (bulk force under the external force field) increases and overcomes the frictional force at the solid-liquid interface (adhesive interfacial force). The primary droplet starts to move downward (see Fig. 2a at *t* = 3000 *τ*).

**Figure 2:**
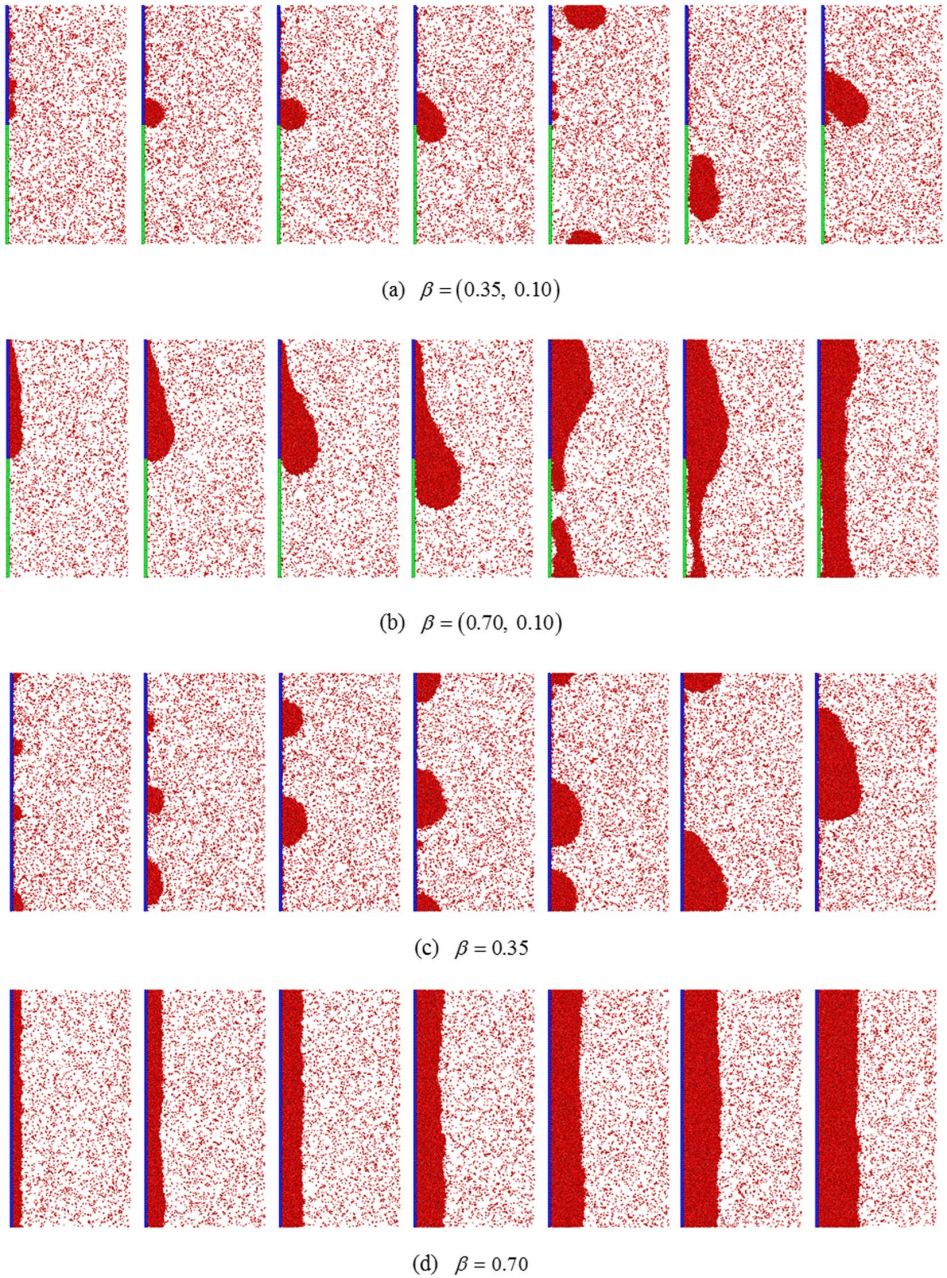
Snapshots at *t* = 1000 *τ*, 2000 *τ*, 3000 *τ*, 4000 *τ*, 5000 *τ*, 6000 *τ* and 7000 *τ* (left to right) in the condensation period.
2. Growing-up and self-shedding
  The primary droplet keeps moving down gradually while growing until it is trapped at the boundary between the condensing and the superhydrophobic surfaces (see Fig. 2a at *t* = 4000 *τ*). This primary droplet is temporarily held by the force balance between the solid-liquid interaction of the condensing surface (adhesive interfacial force), the solid-liquid interaction of the superhydrophobic surface (repulsive interfacial force) and the vertically-driving force on the droplet (bulk force). This primary droplet continues growing up through coalescence with newly-generated clusters as well as condensation of vapor molecules. The bulk force increases as the droplet is upsizing. Eventually, the force balance breaks up and the droplet starts to move downward again (see Fig. 2a at *t *= 5000 *τ*). On entering the superhydrophobic surface, the primary droplet immediately transforms from a cap-like shape to a round shape due to surface tension of the droplet. This instantaneous transformation is associated with the release of the surface free energy of the droplet, which is converted into kinetic energy. The droplet is driven to ‘bounce and fall’, i.e. self-shedding, by the resultant force of the external force downward and the bouncing force normal to the solid surface (see upper panel of Fig. 1b).
3. Falling and sweeping
  After self-shedding, the primary droplet, as a freely falling body, starts to fall acceleratingly under the external force field. Note that there are plenty of ongoing nucleation, coalescences and growing droplets on the condensing surface downstream. Consequently, there is a huge probability for this falling droplet to contact with growing clusters and droplets. Since the velocity component normal to the solid surface, outward, is much smaller than the velocity component parallel to the solid surface, downward, this falling droplet can readily merge with and entrain the growing clusters and droplets by inertia once the contact occurs. The falling droplet keeps upsizing through the mergences. Meanwhile, this falling and entrainment equivalently sweeps the condensing surface downstream. On the other hand, the mergence acts the falling droplet a force to the solid surface, which maintains the merged droplet adhering to the solid surface while falling (see Fig. 2a at *t* = 6000~7000 *τ*). Note that with increasing size and velocity of the falling droplet and adherence to the solid surface, the sweeping effect is continuously enhanced during falling. (See Supplementary Video S1 for animation).

### Filmwise condensation on surface with β = (0.70, 0.10)

1. Growing-up and self-shedding
  The fluid-solid interaction is sufficiently strong so that numerous clusters instantaneously form on the solid surface immediately when the vapor molecules contact the solid surface. A film-like condensate quickly emerges and covers the whole condensing surface, then develops into a complete condensate film and continues to grow thicker. The film thickness is initially uniform and steady (see Fig. 2b at *t *= 1000 *τ*). With increasing thickness, the film gradually feels the external force field and shapes vertically-uneven (see Fig. 2b at *t *= 2000 *τ*). A drop-like condensate appears at the boundary between the condensing surface and the superhydrophobic surface. With condensation ongoing, the drop-like part grows more quickly than the rest due to the accumulation of condensate driven by the external force field (see Fig. 2b at *t *= 3000 *τ*). Finally, the force balance breaks up and the drop-like condensate starts to move downward (see Fig. 2b at *t *= 4000 *τ*). On entering the superhydrophobic surface, the drop-like condensate apparently accelerates and self-sheds with negligible friction due to very weak solid-liquid interaction.
2. Falling and sweeping
  After self-shedding, the drop-like condensate, driven by the external force field, starts to fall acceleratingly. This falling elongates and thins or even breaks the condensate film due to liquid-vapor surface tension, which weakens the pulling effect and favors the acceleration (see Fig. 2b at *t *= 5000~6000 *τ* and lower panel of Fig. 1b). When the falling condensate runs into a growing condensate downstream, they instantly merge and continue falling downward while adhering to the solid surface. The falling decelerates to a certain extent by the mergence while accelerates again when passing the superhydrophobic surfaces. The periodic disturbance of deceleration and acceleration drives the falling condensate to be dynamically wavy (see Fig. 2b at *t* = 7000 *τ*). In addition, these dynamics equivalently give a sweeping effect on the condensing surface. (See Supplementary Video S2 for animation).
  For comparison, we also simulated the condensation on surfaces with uniform values of *β*. In the DWC on surface with *β *= 0.35, the nucleation and coalescence are similar as in the case with *β *= (0.35,0.10). Once the primary droplets form, they keep moving downward on the solid surface and growing while incorporating the clusters and droplets. Due to the friction at the solid-liquid interface, the droplet moving is much slower than that in the case with *β *= (0.35,0.10) (see Fig. 2c). In FWC on a surface with *β *= 0.70, the initial film-like condensate, covering the whole solid surface, keeps growing uniformly. The film starts to move downward when it is sufficiently thick. Due to the frictional force, the film moving is slow and steady (see Fig. 2d). No self-shedding and sweeping are seen in the condensation on these two surfaces with uniform values of *β*. (See Supplementary Videos S3 and S4 for animation).

## Discussion

**Figure 3:**
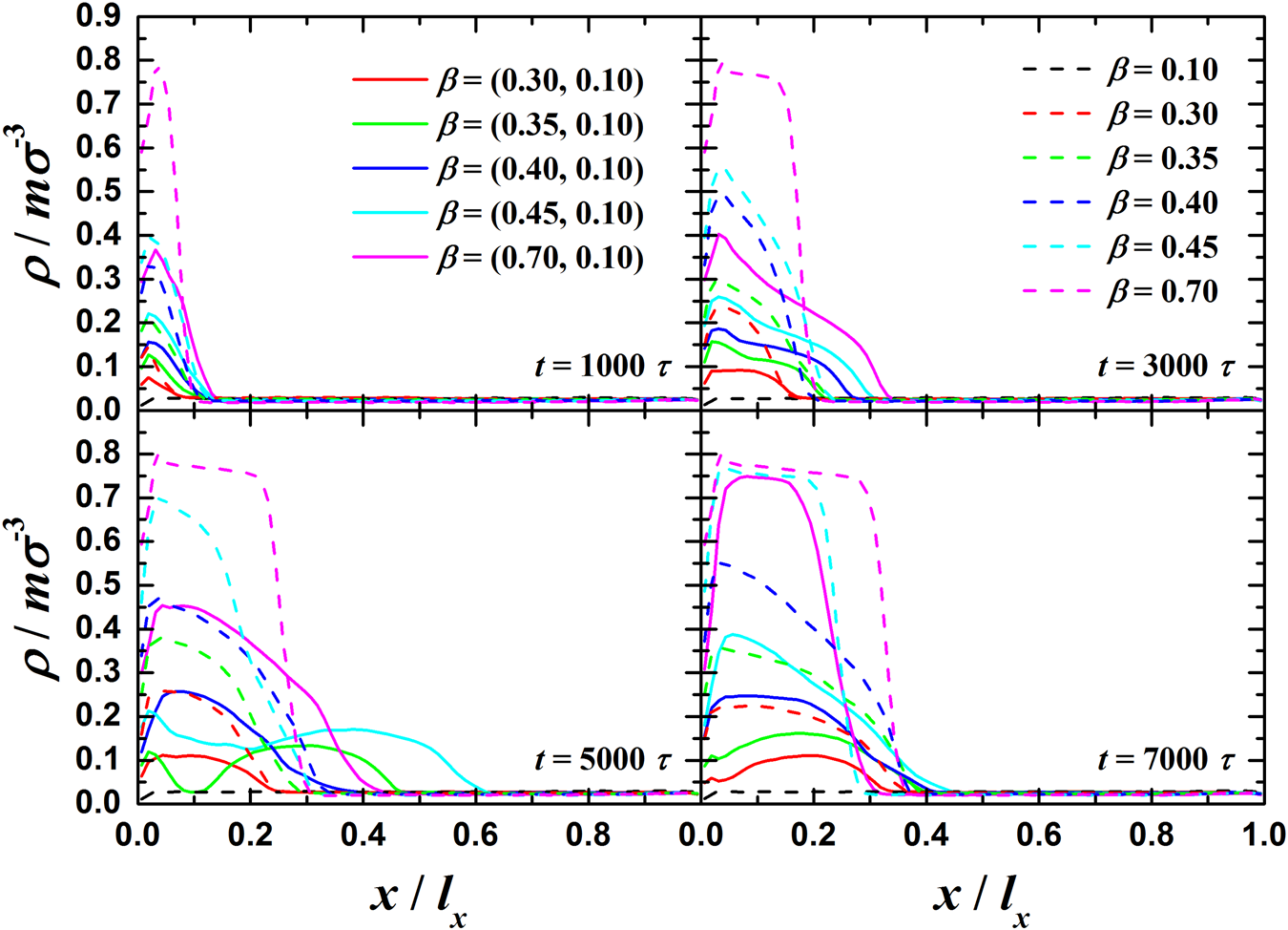
Transient profiles of the averaged density (*ρ*) in *y-z* plane at different times in the condensation period.

The transient profiles of the averaged density in the *x*-direction are given in Fig. 3. We can clearly see that the averaged density of dual-*β* cases is apparently lower than that of uni-*β* cases, which suggests a thinner average condensate in dual-*β* cases. In our recent work, we have shown that the condensation mode is decided by the surface wettability at the onset of surface condensation^18^. Therefore, the differences in condensate growth are due to the dynamics of condensate after the onset. We find that the condensate gradually grows thicker with time for uni-*β* cases and the density monotonically decreases along the *x*-direction in a regular way. Note that the low values adjacent to the solid surface is due to the density oscillation by liquid layering for surfaces with higher *β* (*β *≥ 0.35)^30–32^ while due to the droplet geometry for surfaces with lower *β* (*β *< 0.35). However, the condensate grows diversely in a random way for dual-*β* cases. For example, in the dual-*β* cases with *β *= (0.35,0.10) and *β *= (0.45,0.10), the density profiles apparently show droplets existing near the condensing surface (*x*/*l*_*x*_ = 0.1~0.2) at *t *= 5000 *τ* while the droplets disappears at *t *= 7000 *τ*. These emergence of density drops is due to the detachment of the self-shedding droplets (see Fig. 2a at *t *= 5000 *τ*) and the disappearance is due to the adherence of falling droplets to the solid surface by continuous mergences (see Fig. 2a at *t *= 7000 *τ*). Sometimes, the droplets keep adhering to the solid surface while growing therefore the above-mentioned density drops cannot be seen, e.g. the dual-*β* cases with *β *= (0.30,0.10) and *β *= (0.40,0.10). For FWC, e.g. the dual-*β* case with *β *= (0.70,0.10), the thickness of the condensate largely varies with time but the mean density always remains lower than that of the uni-*β* counterpart due to continuous disturbance by self-shedding and sweeping (see Fig. 2b).

**Figure 4:**
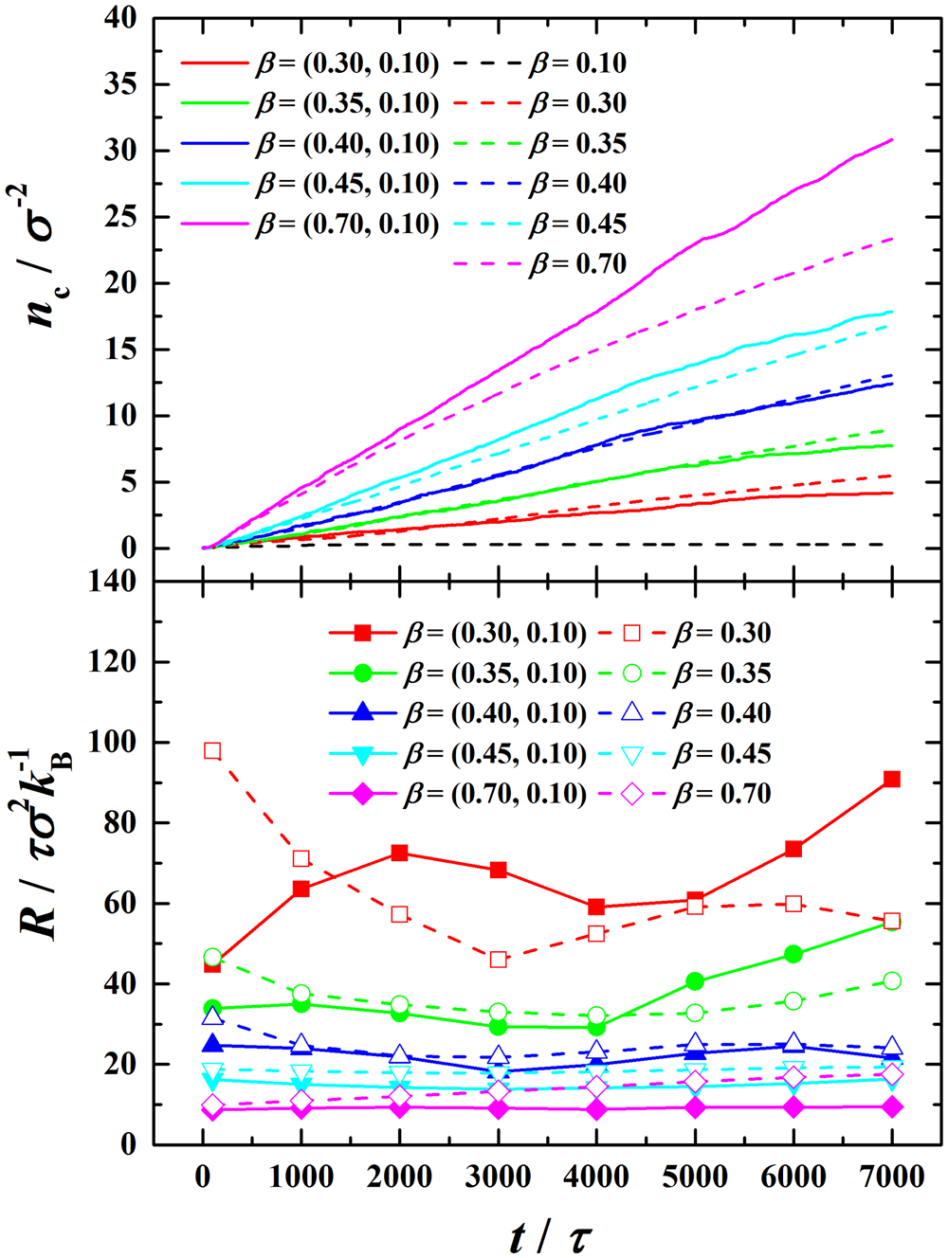
Number of condensed molecules per unit condensing surface area (${n}_{c}$) and thermal resistance (*R*) vs. time (*t*) in the condensation period.

To quantitatively evaluate the heat transfer performances of the dual-*β* and uni-*β* cases, the number of the molecules condensed per unit area of the condensing surface (*n*_c_) with time is recorded (see upper panel of Fig. 4). Generally, the results show monotonically increasing *n*_c_ with time, which means the condensation is continuously occurring at all times, except the near-zero curve for *β *= 0.10 meaning no condensation occurs. Specifically, with increasing *β*, the curve changes from linearity to sub-linearity for the uni-*β* cases. This indicates that the condensation mass flux remains almost constant with time in DWC while it decreases with time in FWC. On the other hand, diverse trends are seen between the dual-*β* and uni-*β* cases. The condensation intensity for dual-*β* cases is superior to that for uni-*β* cases when *β* 0.40 while inferior when *β *< 0.40. *β *= 0.40 is seen to be a threshold. This suggests that only when *β* 0.40 does the composite nano-surface enhance the condensation heat transfer compared to the uni-*β* counterpart. In addition, the enhancement of condensation heat transfer increases with time.

**Figure 5:**
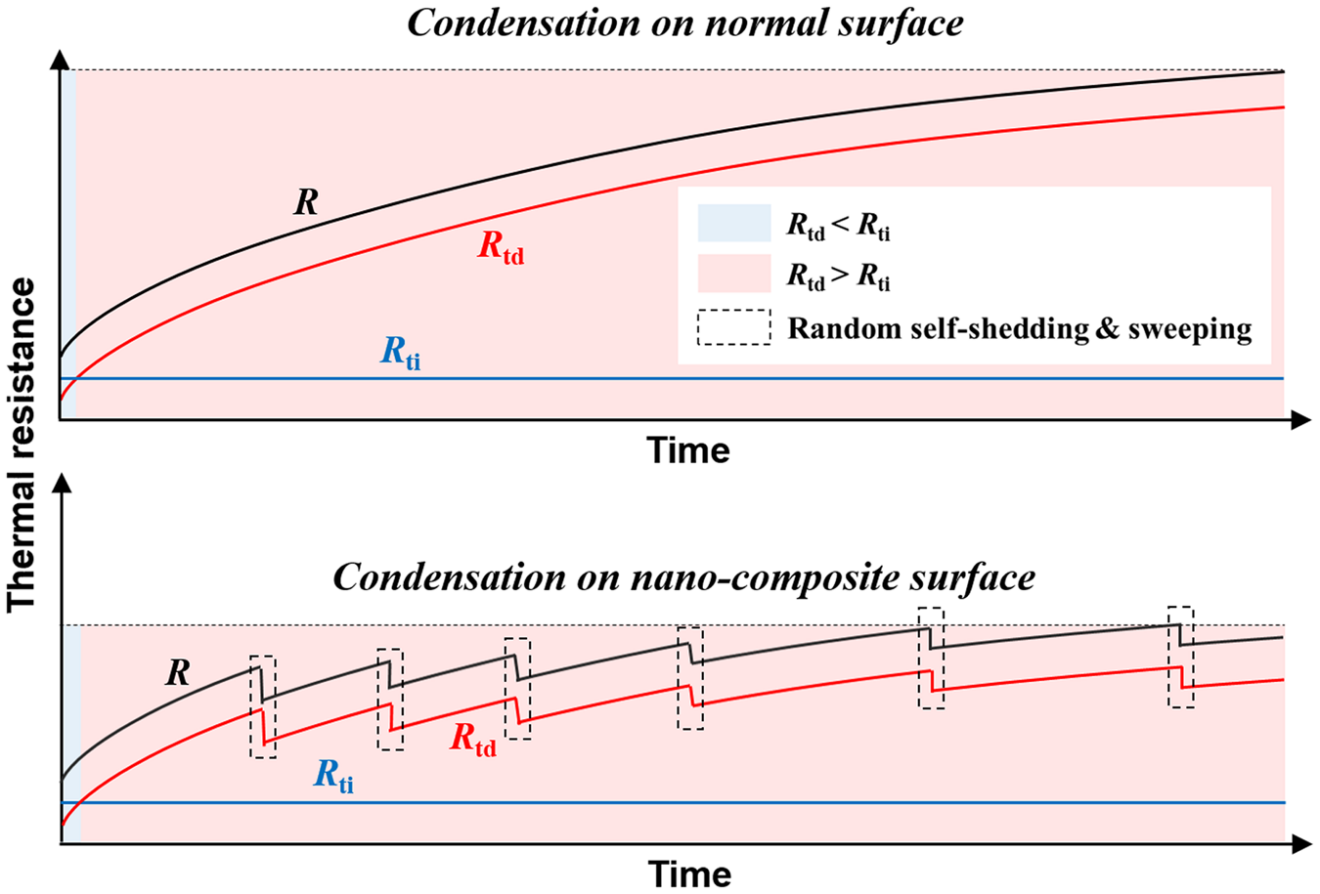
Schematic presentation of the mechanism of self-shedding and sweeping on composite nano-surface to enhance condensation heat transfer.

To reveal the enhancement mechanism underneath, the total thermal resistance is calculated as *R* = Δ*T*/*q*, where Δ*T* = *T*_v_ − *T*_s_ is the vapor-to-solid temperature difference and *q* is the condensation heat flux. *q* is further calculated as $q=({h}_{{\rm{v}}}-{h}_{{\rm{l}}}){\dot{n}}_{{\rm{c}}}$, where *h*_v_ and *h*_l_ are the specific enthalpy in vapor and liquid bulks, respectively (see details in ref. 33); ${\dot{n}}_{{\rm{c}}}$ is the condensation mass flux, i.e. the time derivative of *n*_c_. We find that *R* generally decrease as *β* increases and varies diversely with time in different cases (see lower panel of Fig. 4). When *β* is small, *R* varies drastically with time in the cases of dual-*β* (*β* = (0.30,0.10) and (0.35,0.10)) and uni-*β* (*β* = 0.30 and 0.35). When *β* is intermediate, *R* basically varies similarly in the cases of dual-*β* (*β* = (0.40,0.10)) and uni-*β* (*β* = 0.40). These indicate that the heat transfer enhancement of dual-*β* cases is unconspicuous for small and intermediate *β*. When *β* is large, *R* in the dual-*β* cases (*β* = (0.45,0.10) and (0.70,0.10)) grows apparently much more slowly than that in the uni-*β* cases (*β* = 0.45 and 0.70), indicating steady and conspicuous heat transfer enhancement. In fact, the total thermal resistance (*R*) is comprised of several components, i.e. the solid-liquid interfacial thermal resistance (*R*_sl_), the condensate bulk thermal resistance (*R*_l_), the liquid-vapor interfacial thermal resistance (*R*_lv_) and the curvature-induced thermal resistance (*R*_curv_)^4^. Obviously, the components that vary with the growth of condensate bulk are time-dependent while those only related to the material and fluid properties are time-independent. Therefore, we further categorize them into the time-dependent components (*R*_td_), including *R*_l_ and *R*_curv_, and time-independent components (*R*_ti_), including *R*_sl_ and *R*_lv_. Our recent work has revealed that the competition between *R*_td_ and *R*_ti_ plays an important role throughout the condensation process^19^. Normally, *R*_ti_ dominates the condensation intensity in the initial period of time, whereas, with the condensate bulk growing, *R*_td_ gradually surpasses *R*_ti_ and finally dominates. The proposed composite nano-surface, that can sustain and enhance the condensation heat transfer, is essentially a resort that suppresses *R*_td_ by continuous self-shedding and sweeping of condensate. The enhancement mechanism is schematically illustrated in Fig. 5. Based on this mechanism, the unconspicuous heat transfer enhancement for dual-*β* cases with small and intermediate *β* is due to the dominating *R*_ti_ in the initial period of time, which is irrelevant to the dynamics of condensate.

With the fast-developing micro/nano-machining technology^6, 27, 28, 34^, the artificially-engineered composite nano-surface could potentially be used to freely manipulate condensation mode, by customizing the condensing surface, and intensity, by controlling the external force field. Note that the external force field could be gravitational, electrostatic, magnetic or combination for various occasions. This work also has potential for wide applications such as power plant, air-conditioning, refrigeration, electronic cooling.

In summary, we have demonstrated that the condensation heat transfer can be enhanced on the composite nano-surface under an external force field in both dropwise and filmwise condensation modes. We use the hydrophilic or neutral surface for condensation heat transfer and use the superhydrophobic surface for enhancement by self-shedding and sweeping of condensate. The enhancement mechanism is revealed as the timely suppression over the growing condensate bulk thermal resistance. The suppression is actualized through the self-shedding and sweeping of condensate driven by the competition between interfacial and bulk forces and the release of surface free energy of droplet.

## Methods

We use molecular dynamics (MD) simulation to carry out the investigation. The fluid-fluid interaction is governed by the Lennard-Jones (L-J) potential function $\phi (r)=4\varepsilon [{(\sigma /r)}^{12}-{(\sigma /r)}^{6}]$, where *r* is the intermolecular separation, *σ* and *ε* are the length and energy scales, respectively^35^. The function is truncated at the cut-off radius *r*_c_ = 4.0 *σ*, beyond which the interactions are ignored. The fluid-solid interaction is also described by the L-J potential function but with length scale *σ*_fs_ = 0.91 *σ* and energy scale *ε*_fs_ = *β ε*, where the parameter *β* measures the relative strength of fluid-solid bonding.

The vertical, composite nano-surface is arranged leftmost in the simulation box and represented by three layers of solid molecules forming a (111) plane of a face-centered cubic lattice with the lattice constant *σ*_s_ = 0.814 *σ*. Neighboring solid molecules are connected by Hookean springs with the constant *k* = 3249.1 *εσ* ^−2^ ^36^. Two extra layers of solid molecules are set to the left of the three layers. The left layer is stationary as a frame while the right is governed by the Langevin thermostat $\frac{{\rm{d}}{{\bf{p}}}_{i}}{{\rm{d}}t}=-\alpha {{\bf{p}}}_{i}+{{\bf{f}}}_{i}+{{\bf{F}}}_{i}$, where *p*_*i*_ is the momentum of the *i*th solid molecule, *α* = 168.3*τ* ^−1^ is the damping constant^37^, *f*_*i*_ is the sum of the forces acting on the *i*th solid molecule, *F*_*i*_ is a random force, of which each component is sampled from the Gaussian distribution with zero mean value and variance 2*αk*_B_ *T*_s_/*δt* (*δt* = 0.002 *τ* is the time step, where $\tau =\sqrt{m{\sigma }^{2}/\varepsilon }$ is the time scale, *m* being the mass of a fluid molecule)^36, 37^.

In each run, a relaxation period of 200 *τ* is used to keep the vapor saturated at $T=1.0\,\varepsilon {k}_{{\rm{B}}}^{-1}$, followed by the condensation period of 7000 *τ* with solid temperature at ${T}_{{\rm{s}}}=0.75\,\varepsilon {k}_{{\rm{B}}}^{-1}$ and vapor temperature at ${T}_{{\rm{v}}}=1.0\,\varepsilon {k}_{{\rm{B}}}^{-1}$, respectively. Extra vapor molecules are supplied through the rightmost supply region (thickness ${l}_{x}/10$) by the USHER algorithm^38^ immediately when the density within the supply region is lower than its initial saturation value. During the condensation period, the temperature in the supply region is controlled at ${T}_{{\rm{v}}}=1.0\,\varepsilon {k}_{{\rm{B}}}^{-1}$ by the Langevin thermostat^22, 39^.

## Electronic supplementary material

Supplementary Information

Supplementary Video S1

Supplementary Video S2

Supplementary Video S3

Supplementary Video S4
