# Supplementary material for: Self-shedding and sweeping of condensate on composite nano-surface under external force field: enhancement mechanism for dropwise and filmwise condensation modes
Source: Sci Rep. 2017 Aug 17;7:8633. doi: 10.1038/s41598-017-09194-1 (PMC5561255; doi:10.1038/s41598-017-09194-1)
Supplement: Supplementary file 1 — Supplementary Information [file 41598_2017_9194_MOESM1_ESM.doc]

# Self-shedding and sweeping of condensate on composite nano-surface under external force field: enhancement mechanism for dropwise and filmwise condensation modes

# Jie Sun^ab^**[[1]](#footnote-2)*** and Hua Sheng Wang^c^

^a^*School of Chemical Engineering and Technology, Xi’an Jiaotong University, Xi’an 710049, China*

^b^*Institute of Engineering Thermophysics, Chinese Academy of Sciences, Beijing 100190, China*

^c^*School of Engineering and Materials Science, Queen Mary University of London, London E1 4NS, UK*

**Video captions**

**Video 1:** Snapshots of condensation on the dual-$\beta$ surface with $\beta=\left(0.35,\text{ 0.10}\right)$

**Video 2:** Snapshots of condensation on the dual-$\beta$ surface with $\beta=\left(0.70,\text{ 0.10}\right)$

**Video 3:** Snapshots of condensation on the uni-$\beta$ surface with $\beta=0.35$

**Video 4:** Snapshots of condensation on the uni-$\beta$ surface with $\beta=0.70$

1. ^^Author to whom correspondence should be addressed. Electronic mail: sunjie@mail.etp.ac.cn (J Sun). [↑](#footnote-ref-2)
