# Supplementary figures and images for: Self-shedding and sweeping of condensate on composite nano-surface under external force field: enhancement mechanism for dropwise and filmwise condensation modes
Source: Sci Rep. 2017 Aug 17;7:8633. doi: 10.1038/s41598-017-09194-1 (PMC5561255; doi:10.1038/s41598-017-09194-1)

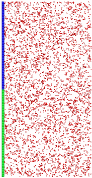

Supplement: Supplementary file 2 — Supplementary Video S1 [file 41598_2017_9194_MOESM2_ESM.gif]

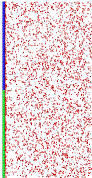

Supplement: Supplementary file 3 — Supplementary Video S2 [file 41598_2017_9194_MOESM3_ESM.gif]

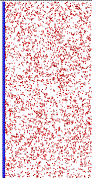

Supplement: Supplementary file 4 — Supplementary Video S3 [file 41598_2017_9194_MOESM4_ESM.gif]

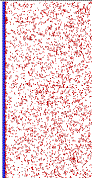

Supplement: Supplementary file 5 — Supplementary Video S4 [file 41598_2017_9194_MOESM5_ESM.gif]
